# *Asterias pectinifera*-Derived Collagen Peptides Mixed with *Halocynthia roretzi* Extracts Exhibit Anti-Photoaging Activities during Exposure to UV Irradiation, and Antibacterial Properties

**DOI:** 10.4014/jmb.2207.07018

**Published:** 2022-10-11

**Authors:** Soo-Jin Oh, Ji-Ye Park, Bada Won, Yong-Taek Oh, Seung-Chan Yang, Ok Sarah Shin

**Affiliations:** 1 BK21 Graduate Program, Department of Biomedical Sciences, College of Medicine, Korea University Guro Hospital, Seoul 08308, Republic of Korea; 2 R&D Center, Star's Tech Co., Ltd., Seoul 08389, Republic of Korea

**Keywords:** *Asterias pectinifera*-derived collagen peptides mixed with *Halocynthia roretzi* extracts (AH), Ethosome-encapsulated AH (E(AH)), antioxidant, anti-photoaging, anti-inflammatory, antibacterial

## Abstract

*Asterias pectinifera*, a species of starfish and cause of concern in the aquaculture industry, was recently identified as a source of non-toxic and highly water-soluble collagen peptides. In this study, we investigated the antioxidant and anti-photoaging functions of compounds formulated using collagen peptides from extracts of *Asterias pectinifera* and *Halocynthia roretzi* (AH). Our results showed that AH compounds have various skin protective functions, including antioxidant effects, determined by measuring the scavenging activity of 2,2-diphenyl-1-picrylhydrazyl radicals, as well as anti-melanogenic effects, determined by measuring tyrosinase inhibition activity. To determine whether ethosome-encapsulated AH compounds (E(AH)) exert ultraviolet (UV)-protective effects, human dermal fibroblasts or keratinocytes were incubated with E(AH) before and after exposure to UVA or UVB. E(AH) treatment led to inhibition of photoaging-induced secretion of matrix metalloproteinase-1 and interleukin-6 and -8, which are associated with inflammatory responses during UV irradiation. Finally, the antibacterial effects of AH and E(AH) were confirmed against both gram-negative and gram-positive bacteria. Our results indicate that E(AH) has the potential for use in the development of cosmetics with a range of skin protective functions.

## Introduction

*Asterias pectinifera* is a starfish species known for its tenacious vitality. It is considered a harmful species as it is capable of consuming various marine organisms, including fish and seaweed, resulting in ecosystem destruction. Furthermore, *Asterias pectinifera* has rapidly increased in number over the last 10 years, thereby contributing to a species diversity collapse in the marine environment. Despite these concerns, several reports have suggested that components derived from these starfish have anti-cancer, antioxidant, anti-inflammatory, neuroprotective, tyrosinase inhibitory, and anti-aging properties [1-5].

We recently reported the conjugation of starfish-derived collagens with a liposome drug delivery system, and *Asterias pectinifera*-derived collagen peptides-encapsulating nanoliposomes showed a high skin absorption rate [6]. Therefore, it is highly likely that *Asterias pectinifera*-derived collagen has the potential to be developed as a cosmetic and pharmaceutical product. Along with extracts of *Asterias pectinifera*, those of sea squirt *Halocynthia roretzi* have also been reported as having broad-spectrum bioactivities, including antioxidant, anti-diabetic, anti-cancer, anti-inflammatory, and anti-microbial effects [7-14]. However, the effects of *Halocynthia roretzi* extracts on skin aging remain to be determined.

Skin aging is caused by both intrinsic chronological aging and extrinsic factors which may be induced by environmental stresses such as smoking and ultraviolet (UV) radiation; UV-induced skin aging is also referred to as photoaging [15-18]. UV can be categorized into UVA, which emits radiation at 320–400 nm, UVB, at 280–320 nm, and UVC, at 100–280 nm [19]. UVA can interpenetrate the human dermal layer of the skin tissue, while UVB can interpenetrate the epidermal layer. Exposure to UV promotes the breakdown of collagen in the skin and disrupts the extracellular matrix (ECM) structure, leading to skin wrinkling. Therefore, skin photoaging can be characterized by decreased collagen and increased matrix metalloproteinase (MMP) expression levels, which can catalyze the ECM as a proteinase, together with increased inflammatory cytokine and chemokine secretion levels [20].

In this study, we prepared ethosome-encapsulated, *Asterias pectinifera*-derived collagen peptides mixed with *Halocynthia roretzi* extracts (E(AH)) and investigated their antioxidant, anti-inflammatory, and anti-photoaging effects using a human primary skin cell model. Additionally, we evaluated the antibacterial effects of *Halocynthia roretzi* extracts, AH compounds, and E(AH) against *Staphylococcus aureus*, *Escherichia coli*, and *Pseudomonas aeruginosa*, some of which are common pathogens known to cause skin infection.

## Materials and Methods

### Preparation of *Asterias pectinifera*-Derived Collagen Peptides Mixed with *Halocynthia roretzi* Extracts (AH)

The extraction of collagen peptides from *Asterias pectinifera* was described in our previous work [6]. *Asterias pectinifera* was briefly washed with ethanol (Duksan Co., Korea) and distilled water. Next, it was incubated in a 12.5% KOH solution to remove non-collagen substances overnight, and the supernatant containing non-collagen substances was removed to collect the bone fragments of *Asterias pectinifera*. Collected bone fragments were then placed in 0.25% tartaric acid (Duksan Co.) to purify the collagen that was attached to the bone fragments. Furthermore, the bone fragments were sonicated at 38 kHz for 1 h to extract the collagen at 4°C by using an HD4400 Ultrasonicator (Bandelin, Germany). Sonicated and extracted collagens were treated with 0.1% protease to break down collagens into peptides and centrifuged at 4,000 x g for 30 min at 4°C to collect a pure collagen solution. Subsequently, the solution was frozen at -80°C and freeze-dried for conversion into a *Asterias pectinifera*-derived collagen peptide powder.

*Halocynthia roretzi* extracts were prepared as previously described, with minor modifications [14]. In summary, *Halocynthia roretzi* extracts were washed with ethanol and distilled water, and the hemolymph was collected. The hemolymph was then incubated with 25 mg ethylenediaminetetraacetic acid (EDTA) powder (Duksan) and centrifuged at 4,000 ×*g* for 30 min at 4°C. The pellet was resuspended in 10 ml 5% acetic acid (Duksan) and sonicated at 25 kHz for 15 s. Next, 40 ml of 5% acetic acid was added to the sonicated solution and stirred overnight at 4°C. Subsequently, the solution was centrifuged to collect the supernatant, followed by gel filtration using a Sephadex G-50 column (Sigma–Aldrich, USA).

### Preparation of Ethosome-Encapsulated AH (E(AH))

E(AH) compounds were generated using the thin-film hydration method. L-α-Phosphatidylcholine (Sigma-Aldrich), TEGO Care CG 90 surfactant (Evonik Industries AG, Germany), and AH compounds were added to 20 ml ethanol (Duksan Co.). We used a rotary evaporator (RE100-Pro, DLAB, China) to completely remove ethanol and form a lipid membrane on the flask wall. The lipid membrane was hydrated in 20 mL 5% ethanol and used as an elastic ethosome. After homogenization to produce a particle with a consistent size using a microfluidizer (M110EH, Microfluidics, USA), the unloaded components were removed using a 0.45 μM syringe filter (Advantec, Japan) and ethosomes were stirred overnight at 4°C for stabilization.

### 1,1 Diphenyl-2 Picrylhydrazyl (DPPH) Assay

For the DPPH assay, 0.1 mM DPPH (Henan Allgreen Chemical Co., Ltd., China) in methanol was prepared. The DPPH solution (800 μl) was mixed with 200 μl of each compound. The mixture was shaken by pipetting and incubated at 23°C for 30 min. The absorbance at 517 nm was measured using an Epoch2 microplate spectrophotometer (BioTek, USA). Ascorbic acid (AA) (Duksan) was used as a positive control. The data were calculated as [100 − (sample value/control value)].

### Mushroom Tyrosinase Inhibition (MTI) Assay

The tyrosinase solution contained 0.1 M phosphate buffer, 1.5 mM tyrosine (Sigma–Aldrich), and 2,000 U/ml tyrosinase (Sigma–Aldrich). Twenty microliters of each compound were added to a 96-well plate, and 280 μl tyrosinase solution was added. The mixture was incubated at 42°C for 15 min and absorbance at 490 nm was measured using a microplate spectrophotometer. The data were calculated as [100-100 × [(sample in tyrosinase buffer-sample in buffer)/(blank in tyrosinase buffer-blank in buffer)].

### Cells

Human keratinocytes (HaCaT) and dermal fibroblasts (HDF) were previously described [17]. HaCaT cells were cultured in Dulbecco’s Modified Eagle Medium (DMEM) (Mediatech Inc., USA) supplemented with 10%fetal bovine serum (FBS) (GenDEPOT, USA), 100 U/ml penicillin, and 100 μg/ml streptomycin (Mediatech Inc.). HDF cells were cultured in RPMI 1640 medium (Mediatech Inc.) supplemented with 10% FBS, 25 mM HEPES, 100 U/ml penicillin, and 100 μg/ml streptomycin. We cultured the cells at 37°C in a 5% CO_2_ incubator and cultured them every 3–4 days.

### Cell Viability Assay

HaCaT and HDF cells were seeded in 96-well plates. After 24 h, the medium was changed, with or without E(AH). At 24 and 48 h, the previous medium was discarded and the 3-(4, 5-dimethyl thiazolyl-2)-2,5-diphenyltetrazolium bromide (MTT; Sigma–Aldrich) was added to the cells and incubated at 37°C in a 5% CO_2_ incubator for 4 h. Dimethyl sulfoxide (DMSO) was added for 30 min, and the absorbance at 570 nm was measured using a Varioskan LUX multimode microplate reader (Thermo Scientific, USA).

### UV Irradiation

UV irradiation was performed as previously described [17]. Cells were seeded in plates and incubated overnight. Before UV irradiation, the cells were washed twice with PBS and the medium was replaced. HDF cells were exposed to UVA (3.0 J/cm^2^) whereas HaCaT cells were exposed to UVB (0.03 J/cm^2^). UVA was supplied with T-8.L 365 nm tubes and UVB was supplied with T-8.M 312 nm tubes using a BioSUN System Illuminator (Vilber Lourmat, France).

### Enzyme-Linked Immunosorbent Assay (ELISA)

MMP-1, *IL-6* and *IL-8* secretion levels from cell supernatant were measured using ELISA kits (R&D, USA). ELISA was performed using the manufacturer’s instructions, and absorbance at 450 nm was measured using a microplate spectrophotometer.

### Antibacterial Assay

The antibacterial assay was performed with methicillin-susceptible *Staphylococcus aureus* (MSSA) (RN6630), methicillin-resistant *Staphylococcus aureus* (MRSA) (MW2), *Escherichia coli* (ATCC 43889, and ATCC 43890), and *Pseudomonas aeruginosa* (PA14) according to the instructions of the Clinical and Laboratory Standards Institute [21].

For the paper disc diffusion assay, LB agar plates were used for *E. coli* growth, and TSB agar plates were used for *S. aureus* and *P. aeruginosa*. After inoculation of bacteria, we placed 8 mm filter paper discs, to which 20 μl of the sample solution was applied, in sterile conditions. Agar plates were incubated at 37°C for 24 h, and then the diameters of the clear zones around the paper discs were measured.

To evaluate the minimum inhibitory concentration (MIC), bacteria were cultured for 24 h in LB or TSB media at 37°C. Then, 100 μl of the adjusted bacterial solution was added to each well of a 96-well plate, followed by the addition of 100 μl of two-fold serial dilutions of the *Halocynthia roretzi* extracts or AH05 compounds to the 96-well plate to obtain concentrations from 1.56 to 100%. The plates were incubated overnight, and the absorbance was measured at 600 nm to observe the antibacterial activity.

To evaluate the antibacterial effects of the compounds, the colony-forming unit assay was performed. AH05 compounds or E(AH) were prepared and added to bacterial suspensions. After overnight incubation at 37°C, the bacterial suspensions were serially diluted and each dilution was plated onto LB or TSB plates. The plates were incubated overnight, and the number of colonies was counted the next day.

### Statistical Analysis

Statistical comparisons between the experimental groups were performed using an unpaired two-tailed Student’s *t*-test or one-way ANOVA (GraphPad Prism), and statistical significance was set at *p* < 0.05.

## Results

### Antioxidant and Tyrosinase Inhibition Effect of AH Compounds

To investigate the cosmeceutical potential of the AH compounds, we extracted collagen from *Asterias pectinifera* and combined it with different ratios of *Halocynthia roretzi* extracts, as previously published [6]. Five different composites of *Asterias pectinifera*-derived collagen peptides relative to the *Halocynthia roretzi* extracts were formulated (Table 1). AH50 represents a 0.5 ratio of *Asterias pectinifera*-derived collagen peptides to *Halocynthia roretzi* extracts, whereas AH01 represents a 0.01 ratio of *Asterias pectinifera*-derived collagen peptides to *Halocynthia roretzi* extracts.

**Fig. 1:**
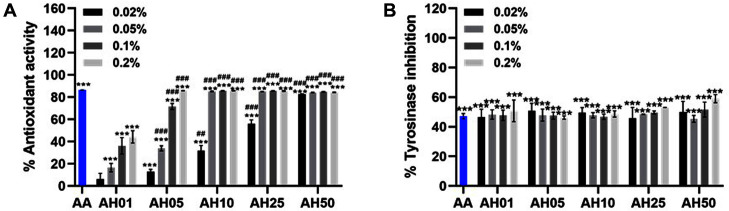
*Asterias pectinifera*-derived collagen peptides mixed with *Halocynthia roretzi* extracts (AH) show antioxidant activity and tyrosinase inhibitory effect. (**A**) To evaluate the extracellular antioxidant level of the AH extracts, the scavenging activities of DPPH radicals were measured with various concentrations of AH extracts. Ascorbic acid (AA) was used as a positive control (1 mg/ml). (**B**) Various concentrations of AH were incubated with the tyrosine and tyrosinase solution and a mushroom tyrosinase inhibition (MTI) assay was performed. AA was used as a positive control (0.3 mg/ml). Values are expressed as the mean ± SD of three determinations. Statistical analysis: ***p < 0.001 vs. control (Ctl); ##*p* < 0.01, ###*p* < 0.001 vs. each concentration of AH01.

First, we examined the antioxidant activities of the AH compounds and performed an extracellular 2,2-diphenyl-1-picrylhydrazyl (DPPH) assay. As shown in Fig. 1A, 0.2% AH (AH05, AH10, AH25, and AH50) showed 85% antioxidant activity, similar to that of ascorbic acid (AA), which was used as a positive control. Along with the antioxidant effect, we also examined tyrosinase activity using a mushroom tyrosinase inhibition (MTI) assay. Tyrosinase is known to play an important role in melanin synthesis, or melanogenesis [22]. While hyperactivation of melanogenesis results in hair, eye, and skin pigmentation, the inhibition of melanin synthesis is linked with a skin-whitening effect [23, 24]. Therefore, the MTI assay represents the skin-whitening effect by evaluating tyrosinase activity. As shown in Fig. 1B, the tyrosinase effect of AH compounds was similar to that of AA, which is a positive control and a well-known tyrosinase inhibitor [25]. Our results showed that different ratios of *Asterias pectinifera*-derived collagen peptides to *Halocynthia roretzi* extracts did not cause significant differences in tyrosinase inhibition. Altogether, these data support the antioxidant and skin-whitening effects of the AH compounds.

### Antioxidant and Tyrosinase Inhibitory Effects of E(AH)

**Fig. 2:**
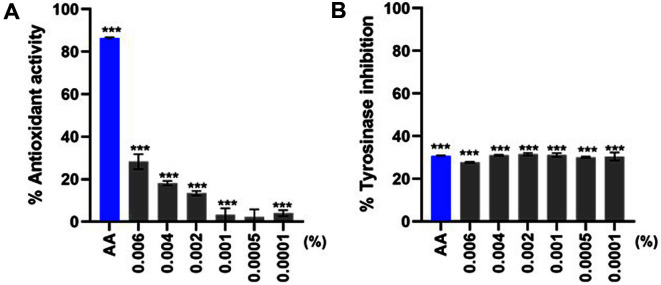
Ethosome-encapsulated AH (E(AH)) shows antioxidant activity and tyrosinase inhibitory effect. (**A**) The scavenging activities of DPPH radicals by E(AH) at various concentrations were measured. Ascorbic acid (AA) was used as a positive control (0.03 mg/ml). (**B**) Mushroom tyrosinase inhibition (MTI) assay was performed using E(AH) compounds. Statistical analysis: ***p < 0.001 vs. Ctl.

To deliver AH compounds more efficiently to skin tissue, we used a conjugated ethosome-based drug delivery system to generate an ethosome-encapsulated *Asterias pectinifera*-derived collagen peptides mixture with *Halocynthia roretzi* extracts (E(AH)), as previously described [6]. Before examining the diverse effects of E(AH) in vitro, we wanted to test the antioxidant and tyrosinase inhibitory effects of E(AH). Figs. 2A and 2B indicated that even at low concentrations E(AH) can still scavenge free radicals and has significant tyrosinase inhibitory activities similar to those of AA.

### Anti-Photoaging Effect of E(AH) Compounds In Vitro

**Fig. 3:**
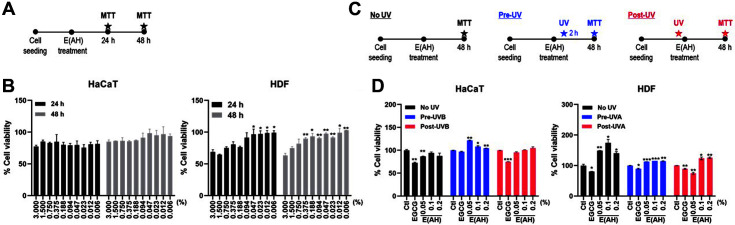
E(AH) treatment does not cause cytotoxicity in UV-irradiated cultured human epidermal keratinocytes (HaCaT) and human dermal fibroblasts (HDF). (**A**) A schematic view of the MTT assay in E(AH)-treated HaCaT and HDF cells. (**B**) HaCaT and HDF cells were treated with various concentrations of E(AH). After incubation, MTT assay was performed to measure the cell viability of E(AH). All data were indicated as mean ± SD of at least three independent experiments. Statistical analysis: *p < 0.05, **p < 0.01. (**C**) Schematic views of the MTT assay in UV-irradiated cells before and after treatment of the E(AH). (**D**) HaCaT and HDF cells were treated with either a control (Ctl), epigallocatechin gallate (EGCG), or various concentrations of E(AH) before or after irradiation with UVA (3.0 J/cm^2^) or UVB (0.03 J/cm^2^). At 48 h, cell viability was examined by MTT assay. EGCG was used as a positive control for the following experiments. All data were indicated as mean ± SD of at least three independent experiments. Statistical analysis: *p < 0.05, **p < 0.01, ***p < 0.001 vs. Ctl-treated cells.

Next, we determined whether E(AH) has an anti-photoaging effect. First, we evaluated the cytotoxicity of the E(AH) treatment on human dermal cells, as shown in Fig. 3A. HaCaT and HDF cells were treated with various concentrations of E(AH) for 24 and 48 h, and cell viability was measured using the MTT assay. We observed decreased cell viability in cells treated with a high concentration of E(AH); however, the 0.094% dose did not show cytotoxicity in either HaCaT or HDF cells (Fig. 3B). Therefore, for the subsequent experiments, 0.05, 0.1, and 0.2%E(AH) doses were used. Furthermore, we examined the viability of UV-irradiated cells before and after treatment with E(AH), as shown in Fig. 3C. HDF cells were irradiated with UVA, whereas HaCaT cells were irradiated with UVB while epigallocatechin-3-gallate (EGCG), a major component of green tea catechin which has anti-cancer properties, was used as a positive control [26, 27]. Pre- or post-UV exposure with various concentrations of E(AH) did not cause a reduction in cell viability, whereas EGCG-treated cells showed a significant reduction in cell viability compared with the control (Fig. 3D).

**Fig. 4:**
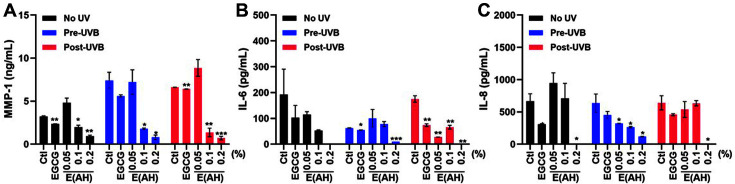
E(AH) treatment in human epidermal keratinocytes (HaCaT) inhibits the secretion of photoaginginduced matrix metalloproteinase-1 (MMP-1) and cytokines. HaCaT cells were treated with a Ctl, EGCG, or various concentrations of E(AH) before or after irradiation with UVB (0.03 J/cm^2^). After 48 h, (**A**) MMP-1, (**B**) *IL-6*, and (**C**) *IL-8* secretion levels in the supernatant were measured by ELISA assay. All data were indicated as mean ± SD of at least three independent experiments. Statistical analysis: *p < 0.05, **p < 0.01, ***p < 0.001 vs. Ctl-treated cells.

**Fig. 5:**
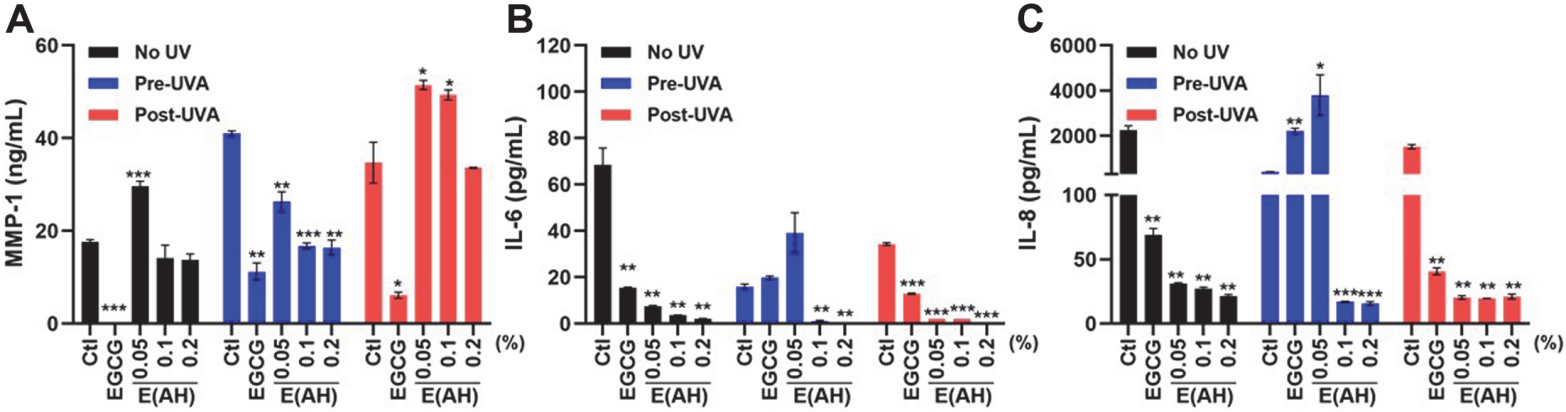
E(AH) treatment in human dermal fibroblasts (HDF) leads to inhibition of photoaging-induced secretion of MMP-1 and cytokines. HDF cells were treated with a Ctl, EGCG, or various concentrations of E(AH) before or after irradiation with UVA (3.0 J/cm^2^). After 48 h, (**A**) MMP-1, (**B**) *IL-6*, and (**C**) *IL-8* secretion levels in the supernatant were measured by ELISA assay. All data were indicated as mean ± SD of at least three independent experiments. Statistical analysis: *p < 0.05, **p < 0.01, ***p < 0.001 vs. Ctl-treated cells.

MMPs are a family of zinc-binding metalloproteinases that catalyze type I, II, and III collagen in the ECM and contribute to the development of disease pathologies, including arthritis and cancer [28, 29]. Furthermore, fragmentation of native fibrillar collagen by MMPs, especially MMP-1, induces skin wrinkling and results in skin senescence. Therefore, MMP-1 can be used as a marker for skin senescence and is classified as a senescence-associated secretory phenotype [18, 20]. Photoaging was induced by UV irradiation in HaCaT and HDF cells, as shown by the increased MMP-1 secretion levels (Figs. 4A and 5A). Treatment with 0.1 or 0.2% E(AH) suppressed UVB-induced MMP-1 secretion to a greater extent than EGCG-treated HaCaT cells (Fig. 4A), whereas E(AH) treatment led to an anti-photoaging effect after UVA exposure in HDF cells (Fig. 5A).

Along with collagenase secretion levels, photoaging-induced *IL-6* and *IL-8* production was measured following E(AH) treatment in UV-exposed cells. As shown in Figs. 4 and 5, *IL-6* and *IL-8* production were significantly impaired by E(AH) treatment in HaCaT and HDF cells. Surprisingly, the secretion of MMP-1, *IL-6*, and *IL-8* was decreased by 10- or 50-fold compared to that in the control cells in 0.2% E(AH)-treated cells. Collectively, these data indicate that E(AH) alleviates UV-induced photoaging and has anti-inflammatory functions.

### Antibacterial Activities of E(AH)

Next, we explored whether AH and E(AH) components would have any antibacterial activity. Gram-negative bacteria, including *E. coli*, are easily spread and may cause food poisoning, and *P. aeruginosa* are typically classified as a hospital-acquired infectious pathogen [30]. Furthermore, antibiotic-resistant bacteria, such as methicillin-susceptible *S. aureus* (MSSA) and methicillin-resistant *S. aureus* (MRSA), threaten public health despite antibiotic development [31].

**Fig. 6:**
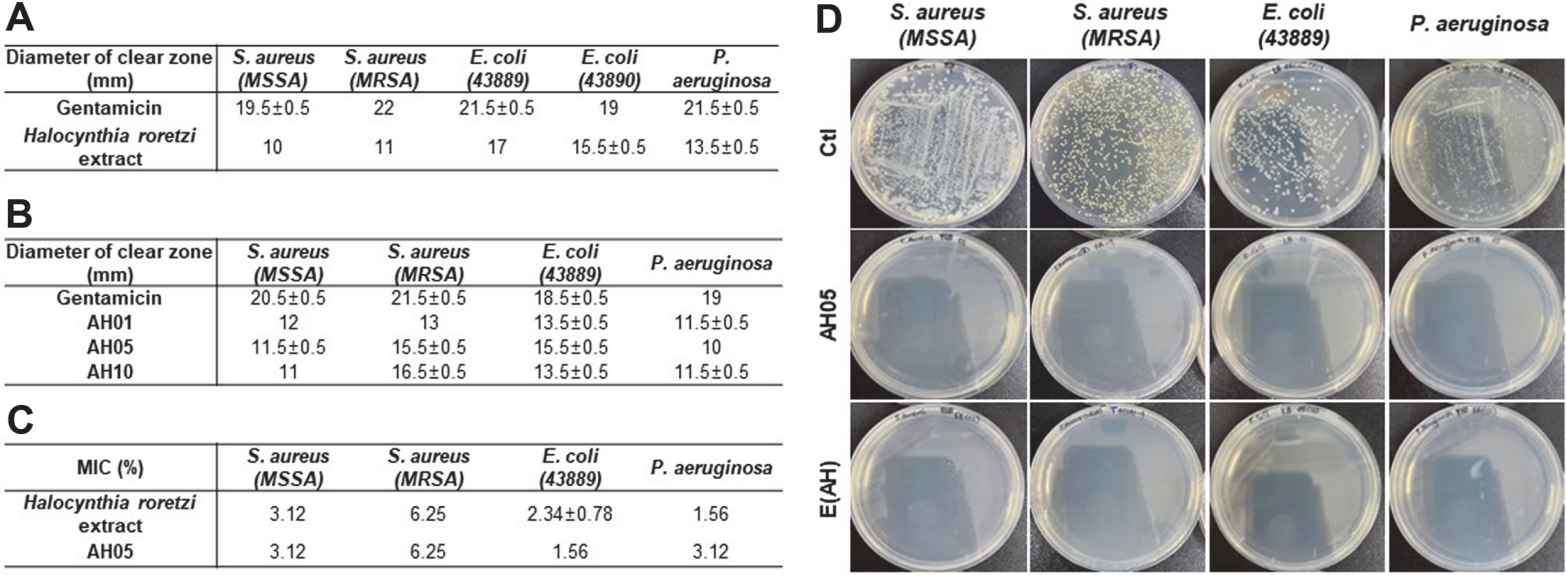
AH and E(AH) show antibacterial activities against multiple bacterial strains. (**A**) For paper disc diffusion assay *S. aureus*, *E. coli*, and *P. aeruginosa* were cultured overnight. Filter paper discs (8 mm) were soaked in *Halocynthia roretzi* extracts and placed on the plate and incubated at 37°C for 24 h. Then, the diameter of the clear zone was measured. (**B**) A paper disc diffusion assay was performed to examine the antibacterial effect of AH compounds against *S. aureus*, *E. coli*, and *P. aeruginosa*. (**C**) Bacteria in the exponential growth phase were inoculated in *Halocynthia roretzi* extracts or AH compoundcontaining media broth. After overnight incubation, absorbance at 600 nm was measured. (**D**) AH05 compound or E(AH) were added to bacterial suspensions of *S. aureus*, *E. coli*, and *P. aeruginosa*. Next day, bacterial suspensions were serially diluted and plated onto the LB or TSB plate to measure the colony-forming units. All data were indicated as mean ± SD of at least three independent experiments.

First, we confirmed the antibacterial effect of the *Halocynthia roretzi* extracts using a paper disc diffusion assay (Fig. 6A). Next, we measured the bacterial growth inhibitory effect of AH compounds against MSSA, MRSA, *E. coli*, and *P. aeruginosa*. As shown in Fig. 6B, AH compounds had an antibacterial effect against these bacteria regardless of the amount of *Asterias pectinifera*-derived collagen peptides. To determine the specific concentration of these compounds that delayed or inhibited bacterial growth, we measured the minimum inhibitory concentration (MIC). As shown in Fig. 6C, *Halocynthia roretzi* extracts and AH05 were able to inhibit the growth of all the bacteria that we tested, with the lowest MIC of 1.56% in our study. Together with these compounds, E(AH) also showed 99.9% antibacterial activities in the colony-forming unit assay (Fig. 6D).

## Discussion

In this study, we investigated the cosmetic potential of collagen peptides extracted from starfish species mixed with sea squirt extracts. *Asterias pectinifera* is widely distributed in Korea and eastern Russia, and along the coastlines of Japan and China. Despite being considered a critical marine pest because of the damage it inflicts on aquaculture fisheries, it is a potential source of numerous bioactive materials, including steroids, saponins, steroidal glycosides, anthraquinones, alkaloids, phospholipids, and peptides, which are involved in diverse biological activities. Here, we report the multi-functional effects of E(AH) on the skin, including the antioxidant, anti-aging, and antibacterial effects, which may make it useful as a pharmaceutical material.

*Halocynthia roretzi*s is considered to have multifunctional biological properties. For example, the vanadium-binding protein and fatty acid extracts from *Halocynthia roretzi* have an anti-diabetic effect by inhibiting adipogenesis [8, 9] and lipopolysaccharide-induced inflammatory responses by targeting nuclear factor kappa B and mitogen-activated protein kinase (MAPK) signaling pathways [10, 32]. In addition, *Halocynthia roretzi* hydrolysates induce apoptosis in cancer cells and exert anti-cancer and antioxidant effects [11, 33]. Furthermore, Plitidepsin (Aplidin) is a drug compound that originated from the Mediterranean sea squirt *Aplidium albicans* and its anti-cancer effects have been proven by several studies [7, 34-36]. Plitidepsin can induce apoptosis in various human cancer cells by targeting the MAPK and epidermal growth factor receptor signaling pathways [7]. Moreover, considering that Plitidepsin shows high antiviral activity against SARS-CoV-2 compared to Remdesivir [13], there may be further reason to investigate the potential of *Halocynthia roretzi* extracts as new antiviral and antibacterial therapeutics.

The body walls of starfish consist of tissues and muscles that are mainly connected by collagens [37-39]. Collagen is a major structural protein that makes skin tissue more resilient and strong, and together with elastin, is widely used in various bio industries such as tissue regeneration. Therefore, efforts have been made to extract collagen from different animals, such as pigs, fish, and cattle, for use as a cosmetic material. However, among collagens from different sources, those isolated from starfish, especially *Asterias pectinifera*, are of lower molecular weight, which results in more efficient delivery and better absorbency into the skin [40-42]. Liposome-mediated transport is considered a novel method for delivering diverse cargos, such as drugs, vaccines, and nucleic acids, to a target region, as they are less cytotoxic and can degrade naturally [43-45]. Ethosomes are nanoliposomes that contain a high proportion of ethanol (20–45%), which differentiates them from regular liposomes [46]. Compared to conventional liposomes, ethosomes can pass through the skin layer, even the epidermis and dermis layer; thus, ethosome delivery shows high permeability and absorbency, especially in the skin. Based on these findings, pharmaceutical delivery systems using ethosomes are in the spotlight, especially with regard to drugs for the skin [47-51]. Our data support that ethosomes serve to overcome the low absorption of collagen peptides into the skin and can be used to maximize the skin permeation of E(AH).

MMPs belong to a family of extracellular zinc-dependent enzymes whose main function is to remodel and degrade the ECM. MMPs induce the cleavage of collagen and elastin, which contribute to skin aging and wrinkle formation. Chronic exposure to UV can cause oxidative damage by inhibiting the activities of endogenous antioxidant enzymes and the accumulation of an abnormal amount of melanin due to increased ROS production. E(AH) treatment may also lead to significant inhibition of the photoaging-induced secretion of MMP-1, *IL-6*, and *IL-8*, which are associated with inflammatory responses during UV irradiation. Regardless of pre- or post-treatment, E(AH) promoted similar skin protective effects, resulting in significant suppression of UV-induced MMP-1 and cytokine production in human skin cells. Given that MMP-1 is an important mediator in UV-irradiated skin damage and the formation of wrinkles, it is important to further delineate the E(AH)-mediated alteration of specific signaling pathways that lead to the reduction of MMP-1 and inflammatory cytokine production.

*S. aureus* colonizes the skin in 20–30% of the population and causes 80–90% of all skin and soft tissue infections in humans worldwide [52-54]. MRSA remains a serious threat to public health owing to its widespread resistance to conventional multidrug antibiotics [55]; thus, it is necessary to investigate novel compounds with promising antibacterial activities against MRSA. Here, we confirmed the antibacterial activities of AH and E(AH) against both common gram-negative and gram-positive bacteria, including MRSA. Given that our data support a broad spectrum of antibacterial activities of AH and E(AH), there is potential for further investigation into the antibacterial role of E(AH) using an in vivo skin infection model.

Taken together, our results suggest that E(AH) is a promising material for the prevention of skin photoaging. Our findings also encourage the further investigation of the effects of E(AH) on skin photoaging in an animal model. Identification and characterization of the active component of E(AH) for anti-inflammatory and antibacterial activities are important, and our current work provides insight into the multi-functional properties of E(AH) for use in the development of novel cosmetics with a broad range of skin protective functions.

## Figures and Tables

**Table 1:** Formulation of *Asterias pectinifera*-derived collagen peptide mixed with *Halocynthia roretzi* extracts (AH).

Sample name	*Asterias pectinifera*-derived collagen peptide (g)	*Halocynthia roretzi* extract (ml)	*Asterias pectinifera*-derived collagen peptide: *Halocynthia roretzi* extract ratio (w/v)
AH01	0.001	0.1	0.01
AH05	0.005	0.1	0.05
AH10	0.01	0.1	0.1
AH25	0.025	0.1	0.25
AH50	0.05	0.1	0.5

## References

[1] Monmai C, Go SH, Shin IS, You S, Kim D-O, Kang S (2018). Anti-inflammatory effect of asterias amurensis fatty acids through NF-kB and MAPK pathways against LPS-stimulated RAW264.7 cells. J. Microbiol. Biotechnol..

[2] Lee CC, Hsieh HJ, Hsieh C-H, Hwang DF (2014). Antioxidative and anticancer activities of various ethanolic extract fractions from crown-of-thorns starfish (*Acanthaster planci*). Environ. Toxicol. Pharmacol..

[3] Zhang W, Wang J, Jin W, Zhang Q (2013). The antioxidant activities and neuroprotective effect of polyAHccharides from the starfish Asterias rollestoni. Carbohydr. Polym..

[4] Jeong MH, Yang KM, Kim JK, Nam BH, Kim GY, Lee SW (2013). Inhibitory effects of *Asterina pectinifera* extracts on melanin biosynthesis through tyrosinase activity. Int. J. Mol. Med..

[5] Thao NP, Cuong NX, Luyen BTT, Quang TH, Hanh TTH, Kim S (2013). Anti-inflammatory components of the starfish *Astropecten polyacanthus*. Mar. Drugs.

[6] Han SB, Won B, Yang SC, Kim DH (2021). *Asterias pectinifera* derived collagen peptide-encapsulating elastic nanoliposomes for the cosmetic application. J. Ind. Eng. Chem..

[7] Cuadrado A, Garcia-Fernandez L, Gonzalez L, Suarez Y, Losada A, Alcaide V (2003). AplidinTM induces apoptosis in human cancer cells via glutathione depletion and sustained activation of the epidermal growth factor receptor, Src, JNK, and p38 MAPK. J. Biol. Chem..

[8] Gunasinghe MA, AT Kim, SM Kim (2019). Inhibitory effects of vanadium-binding proteins purified from the sea squirt *Halocynthia roretzi* on adipogenesis in 3T3-L1 adipocytes. Appl. Biochem. Biotechnol..

[9] Gunasinghe M, SM Kim (2018). Antioxidant and antidiabetic activities of vanadium binding proteins purified from the *Halocynthia roretzi*. J. Food Sci. Technol..

[10] Kim AT, Kim DO (2019). Anti-inflammatory effects of vanadium-binding protein from *Halocynthia roretzi* in LPS-stimulated RAW264.7 macrophages through NF-κB and MAPK pathways. Int. J. Biol. Macromol..

[11] Oh Y, Shim KB, Ahn CB, Kim SS, Je JY (2019). *Halocynthia roretzi* (*Halocynthia roretzi*) hydrolyAHtes induce apoptosis in human colon cancer HT-29 cells through activation of reactive oxygen species. Nutr. Cancer.

[12] Park JH, Seo BY, Lee SC, Park E (2010). Effects of ethanol extracts from stalked *Halocynthia roretzi* (Styela clava) on antioxidant potential, oxidative DNA damage and DNA repair. Food Sci. Biotechnol..

[13] White KM, Rosales R, Yildiz S, Kehrer T, Miorin L, Moreno E (2021). Plitidepsin has potent preclinical efficacy against AHRSCoV-2 by targeting the host protein eEF1A. Science.

[14] Jang WS, Kim KN, Lee YS, Nam MH, Lee IH (2002). Halocidin: a new antimicrobial peptide from hemocytes of the solitary tunicate, *Halocynthia aurantium*. FEBS Lett..

[15] Oh S-J, JK Lee, OS Shin (2019). Aging and the immune system: the impact of immunosenescence on viral infection, immunity and vaccine immunogenicity. Immune Netw..

[16] Lee JK, Oh SJ, Gim JA, Shin OS (2022). miR-10a, miR-30c, and miR-451a encapsulated in small extracellular vesicles are prosenescence factors in human dermal fibroblasts. J. Invest. Dermatol..

[17] Seo SW, Park SK, Oh SJ, Shin OS (2018). TLR4-mediated activation of the ERK pathway following UVA irradiation contributes to increased cytokine and MMP expression in senescent human dermal fibroblasts. PLoS One.

[18] Gilchrest BA (2013). Photoaging. J. Investig. Dermatol..

[19] Collado M, Blasco MA, Serrano M (2007). Cellular senescence in cancer and aging. Cell.

[20] Ghosh K, BC Capell (2016). The senescence-associated secretory phenotype: critical effector in skin cancer and aging. J. Investig. Dermatol..

[21] Wikler M, F Cockerill, W Craig (2006). Performance standards for antimicrobial disc susceptibility tests; Standards.

[22] D'Mello SAN, Finlay GJ, Baguley BC, Askarian-Amiri ME (2016). Signaling pathways in melanogenesis. Int. J. Mol. Sci..

[23] Slominski A, Tobin DJ, Shibahara S, Wortsman J (2004). Melanin pigmentation in mammalian skin and its hormonal regulation. Physiol. Rev..

[24] Qian W, Liu W, Zhu D, Cao Y, Tang A, Gong G (2020). Natural skin-whitening compounds for the treatment of melanogenesis. Exp. Ther. Med..

[25] Pillaiyar T, Namasivayam V, Manickam M, Jung SH (2018). Inhibitors of melanogenesis: an updated review. J. Med. Chem..

[26] Lambert JD, Elias RJ (2010). The antioxidant and pro-oxidant activities of green tea polyphenols: a role in cancer prevention. Arch. Biochem. Biophys..

[27] Bigelow R, Cardelli J (2006). The green tea catechins,(−)-Epigallocatechin-3-gallate (EGCG) and (−)-Epicatechin-3-gallate (ECG), inhibit HGF/Met signaling in immortalized and tumorigenic breast epithelial cells. Oncogene.

[28] Singh D, Srivastava Sk, Chaudhuri Tk, Upadhyay G (2015). Multifaceted role of matrix metalloproteinases (MMPs). Front. Mol. Biosci..

[29] Burrage PS, Mix KS, Brinckerhoff CE (2006). Matrix metalloproteinases: role in arthritis. Front. Biosci..

[30] Catho G, Martischang R, Boroli F, Chraiti MN, Martin Y, Tomsuk ZK (2021). Outbreak of *Pseudomonas aerugino* producing VIM carbapenemase in an intensive care unit and its termination by implementation of waterless patient care. Crit. Care.

[31] Enright MC, Robinson DA, Randle G, Feil EJ, Grundmann H, Spratt B (2002). The evolutionary history of methicillin-resistant *Staphylococcus aureus* (MRAH). Proc. Natl. Acad. Sci. USA.

[32] Monmai C, Go SH, Shin I-S, You SG, Lee H, Kang SB (2018). Immune-enhancement and anti-inflammatory activities of fatty acids extracted from *Halocynthia aurantium* tunic in RAW264.7 cells. Mar. Drugs.

[33] Kim SS, Ahn CB, Moon SE, Je JY (2018). Purification and antioxidant activities of peptides from *Halocynthia roretzi* (*Halocynthia roretzi*) protein hydrolyAHtes using pepsin hydrolysis. Food Biosci..

[34] Delgado-Calle J, Kurihara N, Atkinson EG, Nelson J, Miyagawa K, Galmarini CM (2019). Aplidin (plitidepsin) is a novel antimyeloma agent with potent anti-resorptive activity mediated by direct effects on osteoclasts. Oncotarget.

[35] Gomes NGM, Valentao PB, Pereira RB (2020). Plitidepsin to treat multiple myeloma. Drugs Today (Barc).

[36] LoAHda A, Munoz-Alonso MJ, Garcia C, Sanchez-Murcia PA, Martinzw-L JF, Dominguez JM (2016). Translation elongation factor eEF1A2 is a novel anticancer target for the marine natural product plitidepsin. Sci. Rep..

[37] Blowes LM, Egertova M, Liu Y, Davis GR, Terrill NJ, Gupta HS (2017). Body wall structure in the starfish *Asterias rubens*. J. Anat..

[38] O'Neill P (1989). Structure and mechanics of starfish body wall. J. Exp. Biol..

[39] Eylers JP (1976). Aspects of skeletal mechanics of the starfish Asterias forbesii. J. Morphol..

[40] Park S.-H, Song T, Bae TS, Khang G, Choi BH, Park SR (2012). Comparative analysis of collagens extracted from different animal sources for application of cartilage tissue engineering. Int. J. Precis. Eng. Manufact..

[41] Ikoma T, Kobayashi H, Tanaka J, Walsh D, Mann S (2003). Physical properties of type I collagen extracted from fish scales of *Pagrus major* and *Oreochromis niloticas*. Int. J. Biol. Macromol..

[42] Sun L, Hou H, Li B, Zhang Y (2017). Characterization of acid-and pepsin-soluble collagen extracted from the skin of Nile tilapia (*Oreochromis niloticus*). Int. J. Biol. Macromol..

[43] Sercombe L, Veerati T, Moheimani F, Wu SY, Sood AK, Hua S (2015). Advances and challenges of liposome assisted drug delivery. Front. Pharmacol..

[44] Akbarzadeh A, Rezaei-Sadabady R, Davaran S, Joo SW, Zarghami N, Hanifehpour Y (2013). Liposome: classification, preparation, and applications. Nanoscale Res. Lett..

[45] Zylberberg C, S Matosevic (2016). Pharmaceutical liposomal drug delivery: a review of new delivery systems and a look at the regulatory landscape. Drug Deliv..

[46] Paiva-AHntos AC, Silva AL, Guerra C, Peixoto D, Pereira-Silva M, Zeinali M (2021). Ethosomes as nanocarriers for the development of skin delivery formulations. Pharm. Res..

[47] Nasr S, Rady M, Gomaa I, Syrovets T, Simmet T, Fayad W (2019). Ethosomes and lipid-coated chitoAHn nanocarriers for skin delivery of a chlorophyll derivative: a potential treatment of squamous cell carcinoma by photodynamic therapy. Int. J. Pharm..

[48] ElAHyed MM, Abdallah OY, Naggar VF, Khalafallah NM (2006). Deformable liposomes and ethosomes: mechanism of enhanced skin delivery. Int. J. Pharm..

[49] Bellefroid C, Lechanteur A, Evrard B, Mottet D, Debacq-Chainiaux F, Piei G (2019). In vitro skin penetration enhancement techniques: a combined approach of ethosomes and microneedles. Int. J. Pharm..

[50] Shen LN, Zhang YT, Wang Q, Xu L, Feng NP (2014). Enhanced in vitro and in vivo skin deposition of apigenin delivered using ethosomes. Int. J. Pharm..

[51] Van Tran V, JY Moon, YC Lee (2019). Liposomes for delivery of antioxidants in cosmeceuticals: challenges and development strategies. J. Control. Release.

[52] Tong SY, Davis JS, Eichenberger E, Holland TL, Fowler JR VG (2015). *Staphylococcus aureus* infections: epidemiology, pathophysiology, clinical manifestations, and management. Clin. Microbiol. Rev..

[53] Dayan GH, Mohamed N, Scully IL, Cooper D, Begier E, Eiden J (2016). *Staphylococcus aureus*: the current state of disease, pathophysiology and strategies for prevention. Expert Rev. Vccines.

[54] Peterson LR, DM Schora (2016). Methicillin-resistant *Staphylococcus aureus* control in the 21st century: laboratory involvement affecting disease impact and economic benefit from large population studies. J. Clin. Microbiol..

[55] Choi EJ, Kim HI, Kim JA, Jun SY, Kang SH, Park DJ (2015). The herbal-derived honokiol and magnolol enhances immune response to infection with methicillin-sensitive *Staphylococcus aureus* (MSAH) and methicillin-resistant *S. aureus* (MRAH). Appl. Microbiol. Biotechnol..

